# Setdb1-mediated H3K9 methylation is enriched on the inactive X and plays a role in its epigenetic silencing

**DOI:** 10.1186/s13072-016-0064-6

**Published:** 2016-05-18

**Authors:** Andrew Keniry, Linden J. Gearing, Natasha Jansz, Joy Liu, Aliaksei Z. Holik, Peter F. Hickey, Sarah A. Kinkel, Darcy L. Moore, Kelsey Breslin, Kelan Chen, Ruijie Liu, Catherine Phillips, Miha Pakusch, Christine Biben, Julie M. Sheridan, Benjamin T. Kile, Catherine Carmichael, Matthew E. Ritchie, Douglas J. Hilton, Marnie E. Blewitt

**Affiliations:** The Walter and Eliza Hall Institute of Medical Research, 1G Royal Parade, Parkville, Melbourne, VIC 3052 Australia; Department of Medical Biology, University of Melbourne, Melbourne, VIC 3010 Australia; Department of Mathematics and Statistics, University of Melbourne, Melbourne, VIC 3010 Australia; Department of Genetics, University of Melbourne, Melbourne, VIC 3010 Australia

**Keywords:** H3K9 methylation, X inactivation, Epigenetic silencing, Setdb1

## Abstract

**Background:**

The presence of histone 3 lysine 9 (H3K9) methylation on the mouse inactive X chromosome has been controversial over the last 15 years, and the functional role of H3K9 methylation in X chromosome inactivation in any species has remained largely unexplored.

**Results:**

Here we report the first genomic analysis of H3K9 di- and tri-methylation on the inactive X: we find they are enriched at the intergenic, gene poor regions of the inactive X, interspersed between H3K27 tri-methylation domains found in the gene dense regions. Although H3K9 methylation is predominantly non-genic, we find that depletion of H3K9 methylation via depletion of H3K9 methyltransferase Set domain bifurcated 1 (Setdb1) during the establishment of X inactivation, results in failure of silencing for around 150 genes on the inactive X. By contrast, we find a very minor role for Setdb1-mediated H3K9 methylation once X inactivation is fully established. In addition to failed gene silencing, we observed a specific failure to silence X-linked long-terminal repeat class repetitive elements.

**Conclusions:**

Here we have shown that H3K9 methylation clearly marks the murine inactive X chromosome. The role of this mark is most apparent during the establishment phase of gene silencing, with a more muted effect on maintenance of the silent state. Based on our data, we hypothesise that Setdb1-mediated H3K9 methylation plays a role in epigenetic silencing of the inactive X via silencing of the repeats, which itself facilitates gene silencing through alterations to the conformation of the whole inactive X chromosome.

**Electronic supplementary material:**

The online version of this article (doi:10.1186/s13072-016-0064-6) contains supplementary material, which is available to authorized users.

## Background

Despite the importance of epigenetic silencing for normal development and differentiation, in most instances of gene silencing, we still do not completely understand the molecular mechanisms of epigenetic repression. X chromosome inactivation (XCI), the dosage compensation mechanism in female mammals, is one of the best-characterised epigenetic processes. It provides a powerful system where epigenetic silencing can be studied for hundreds of genes in parallel. Using X inactivation as a model system, gene silencing can be broken down into overlapping phases, including: initiation, where the silent state is induced; establishment, where the silencing signal is converted into transcriptional silencing; and maintenance, where the repressed state is reinforced to ensure mitotic heritability. Each phase of silencing can be monitored in vitro or in vivo as female cells transition from two active X chromosomes to one active (Xa) and one inactive (Xi). In vivo, XCI occurs in the early post-implantation epiblast. In vitro, the same process occurs when female embryonic stem (ES) cells are differentiated.

XCI is initiated by upregulation of the long non-coding RNA X inactive-specific transcript (*Xist*), which coats the future Xi [1, 2]. *Xist* expression rapidly prompts a series of chromatin changes, including loss of histone acetylation [3] and histone 3 lysine 4 tri-methylation [4] on the future Xi. The Xi also accumulates repressive histone marks, including histone 3 lysine 27 tri-methylation (H3K27me3), which is laid down by polycomb repressive complex 2 (PRC2) [5–7], and histone 2A lysine 119 monoubiquitination (H2AK119ub1) by PRC1 [8–11]. Interestingly, while these chromatin changes predominantly occur early in the time course of XCI, at least for H3K27me3 this mark is neither sufficient nor necessary to establish silencing [12] suggesting a gap in our understanding of epigenetic silencing on the Xi.

Relatively late in the ontogeny of XCI, CpG islands of genes subject to X inactivation become methylated [3]. This requires DNA methyltransferase 1 (Dnmt1) and often structural maintenance of chromosomes hinge domain containing 1 (Smchd1) [13], both of which are required for preserving the epigenetically silent state [14, 15].

In addition to the epigenetic modifications described above, histone 3 lysine 9 (H3K9) methylation is more controversially associated with the inactive X, based on a series of immunofluorescence studies. H3K9 di-methylation (H3K9me2) was ostensibly found on the Xi from the earliest phases of XCI [4, 16–18]; however, later work suggested this was due to cross-reactivity of the anti-H3K9me2 antibody with H3K27me3 [6]. H3K9 tri-methylation (H3K9me3) accumulates on the Xi of many species, although H3K9me3 has not been reported as enriched on the murine Xi [19]. Interestingly, recent work from Minkovsky et al. found that the H3K9 methyltransferase SET domain bifurcated 1 (Setdb1) is involved in maintenance of inactivation of an X-linked reporter allele in murine cells, while immunofluorescence with a new antibody showed H3K9me2 enriched on the putative Xi, as it presents as a dense focus that overlaps with H3K27me3 in female mouse cells, most strikingly early in ES cell differentiation [20]. These suggest that H3K9 methylation may indeed be enriched on the Xi and play a role in XCI, but do not provide definitive evidence for the presence of methylation or the extent of its role in silencing of endogenous X-linked genes. Here we report the first genomic analysis of H3K9 di- and tri-methylation across the inactive X chromosome. Our work reveals that Setdb1-mediated H3K9 methylation is enriched at the intergenic regions of the inactive X, yet plays an important role in epigenetic silencing at the genes on this chromosome and indeed genome-wide.

## Results

### H3K9me2 and H3K9me3 are enriched on the inactive X, predominantly at intergenic regions

H3K9 methylation has controversially been associated with the mouse inactive X, based on immunofluorescence studies. These studies require that the inactive X is enriched in H3K9 methylation compared with the autosomes, in order to visualise a more densely staining region in female cells. H3K9 methylation has not been studied using chromatin immunoprecipitation followed by sequencing (ChIP-seq) in female mouse cells, a more sensitive approach which doesn’t require a difference between the inactive X and autosomes, but rather the more functionally relevant comparison between the inactive and active X chromosomes. Therefore, we set out to investigate whether H3K9me2 and H3K9me3 accumulate on the mouse inactive X by ChIP-seq. To enable allele-specific analyses, we produced mouse embryonic fibroblasts (MEFs) from *Mus musculus castaneus* sires mated with *Mus musculusdomesticusXist*^∆A/+^ dams. The *Xist*^*∆A*^ mutant allele [21] ensured the *castaneus* X was on the obligate Xi. The combination of X-linked single nucleotide polymorphisms (SNPs) between *castaneus* and *domesticus* strains and the *Xist*^∆A^ allele allows us to discriminate between the Xa (*domesticus*) and Xi (*castaneus*). We used male cells as an additional control for the active X chromosome. We then performed ChIP-seq for H3K9me2 and H3K9me3, along with H3K27me3.

**Fig. 1:**
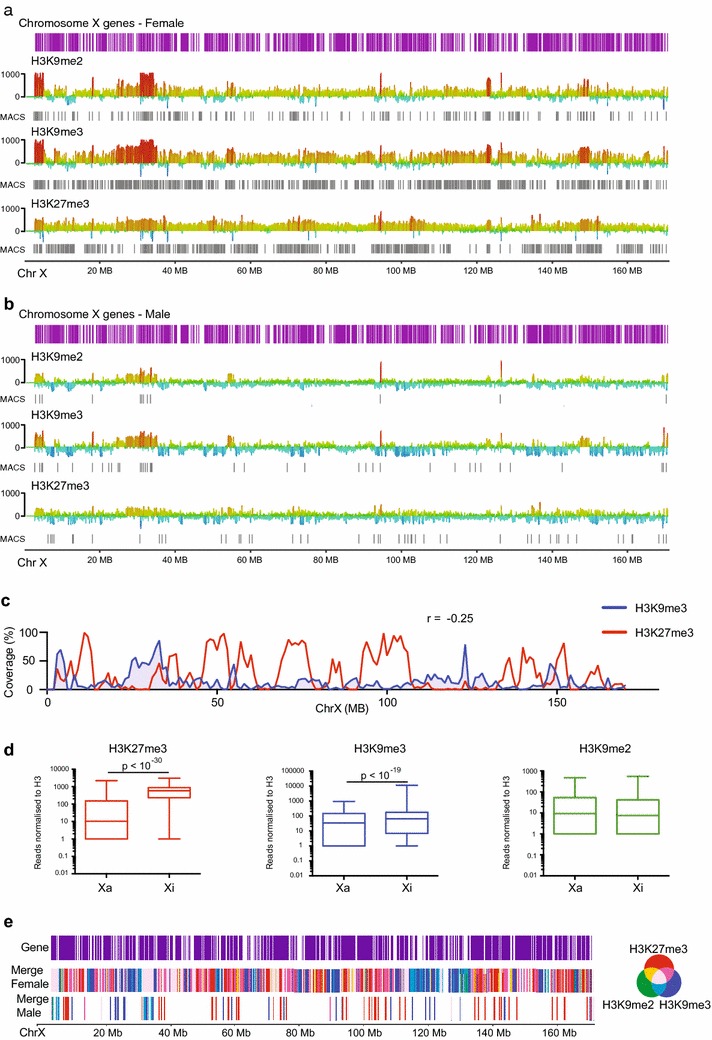
ChIP-seq shows H3K9 methylation is enriched on the inactive X at gene poor regions. Histone ChIP-seq data from 129/CAST F1 *Setdb1* ^+/+^ *Xist* ^∆A/+^ 129/CAST F1 female MEFs transduced with non-silencing (Nons) hairpin (female, n = 3) and 129/CAST F1 *Setdb1* ^+/+^ male MEFs transduced with Nons (male n = 3) MEFs. **a** H3K9me2, H3K9me3 and H3K27me3 ChIP-seq tracks for the entire female X chromosome. *Scale bars* indicate normalised reads relative to H3, where reads above the axis show enrichment and below depletion. Significance of enrichment or depletion is given by the strength of colour—*red* is most enriched, *blue* is most depleted. Location of genes (*purple*), and peaks called using Seqmonk in-built MACS peak caller (*grey*) are indicated. **b** As for **a**, except for male chromosome X. **c** Enrichment analysis, showing percentage coverage of H3K9me3 (*blue*) and H3K27me3 (*red*) ChIP-seq peaks at 50 kb bins along the Female X chromosome. The Pearson correlation coefficient (*r*) is indicated. **d** Number of reads for each histone mark on the Female Xa and Xi, relative to H3. The *box* indicates the first to third quartile, *horizontal line* indicates the median, while whiskers extend to the minimum and maximum values. *p* values shown were determined by Student’s two-tailed paired *t* test. **e** Location of ChIP-seq peaks for H3K27me3 (*red*, n = 3), H3K9me2 (*green* n = 3) and H3K9me3 (*blue* n = 3) shown as a merge in female or male MEFs. Location of genes is indicated (*purple*). A Venn diagram is included to show the colours produced when the various peaks overlap. See also Additional file 2: Figure S2

H3K27me3 is a prominent mark of the Xi that, through a combination of immunofluorescence [5–7] and ChIP-seq, has been shown to distribute in a banded pattern in mouse [22] and human [23, 24]. We also find H3K27me3 occupies the female X chromosome in a banded pattern in MEFs, coincident with gene dense regions (Fig. 1a). As expected, there is far less H3K27me3 enrichment on the male active X (Fig. 1b), consistent with the enrichment found in females being due to the presence of an inactive X chromosome.

H3K9me2 and H3K9me3 also occupy the female X chromosome in a pattern of regions of dense peaks neighbouring regions of sparse peaks, although the pattern is not as clearly defined as H3K27me3. Just as for H3K27me3, far fewer H3K9me2 and H3K9me3 peaks were found on the male active X (Fig. 1b), suggesting the enrichment found in females is caused by H3K9me2 and H3K9me3 accumulation on the Xi. In contrast to H3K27me3, H3K9me3 methylation is predominant in gene poor regions of the X chromosome, often populated by pseudogenes, consistent with previous reports of H3K9me3 at repetitive elements [25] (Fig. 1a). This means H3K9me3 and H3K27me3 occupy predominantly different areas of the female X chromosome (Fig. 1c Pearson correlation *r* = −0.25, Additional file 1: Figure S1a). This pattern of H3K27me3 marking genes and H3K9me3 marking repetitive, gene poor regions is also found on the autosomes (Additional file 1: Figure S1b). To test the enrichment of H3K27me3 at gene-rich regions and H3K9me2 and me3 at pseudogenes, we performed a Pearson correlation analysis and found a positive correlation between H3K27me3 and genes (*r* = 0.37), a positive correlation between H3K9me3 and pseudogenes (*r* = 0.33), but a negative correlation between H3K9me2 and H3K9me3 and genes (*r* = −0.2 and −0.42, respectively) (Additional file 1: Figure S1c). These data confirm that the H3K9 methyl marks are enriched at different regions of the X chromosome in females than H3K27me3.

We next split the ChIP-seq reads according to whether they map to the *castaneus* X (Xi), *domesticus* X (Xa), or those that do not contain informative SNPs. As expected, when considering the H3K27me3 peak regions, we found that H3K27me3 is strongly enriched on the Xi (Fig. 1d). However, we found a far lower density of SNPs in the gene poor regions where H3K9me2 and H3K9me3 peaks are called compared with the genic H3K27me3 peak regions (Additional file 1: Figure S1d). The paucity of informative SNPs in the H3K9 methylation marked regions results in fivefold to tenfold reduction in the average informative H3 reads per peak compared with H3K27me3 peak regions, making the allele-specific analysis challenging. All the same, when the informative reads were taken into account for regions defined as peaks using all reads for H3K9me3, we observe a significant enrichment on the Xi compared with the Xa (Fig. 1d). For H3K9me2 we do not observe a significant enrichment on the Xi (Fig. 1d). It is not clear whether this is due to H3K9me2 marked regions having the lowest density of SNPs of all three histone marks examined (Additional file 1: Figure S1d). Notably though, we observe a threefold increase in both H3K9me2 and H3K9me3 ChIP-seq peaks on the X in females, but not in males (Fig. 4a), suggesting a specific enrichment for both of these marks on the Xi. To confirm these findings, we performed immunofluorescence for H3K9me2 and H3K9me3 in female MEFs. Similar to others [20, 26], we observed clear colocalisation of H3K9me2 with the Xi in 100 % of cells tested. We also observed colocalisation with H3K9me3 and the Xi in 97 % of cells tested, which has not been previously reported (Additional file 2: Figure S2).

Overall, through a combination of comparisons between male and female samples, and our allele-specific analysis, our data support the allele-specific data for H3K9me3 and suggest that H3K9me2 is also enriched on the Xi, despite not being measurable using the SNPs between the *castaneus* and *domesticus* genomes. The interspersed banding pattern of H3K9me3 and H3K27me3 reveals an inactive X chromosome almost completely covered in repressive histone marks (Fig. 1e).

### Setdb1 is the most dose-dependent H3K9 methyltransferase required for maintenance of XCI

**Fig. 2:**
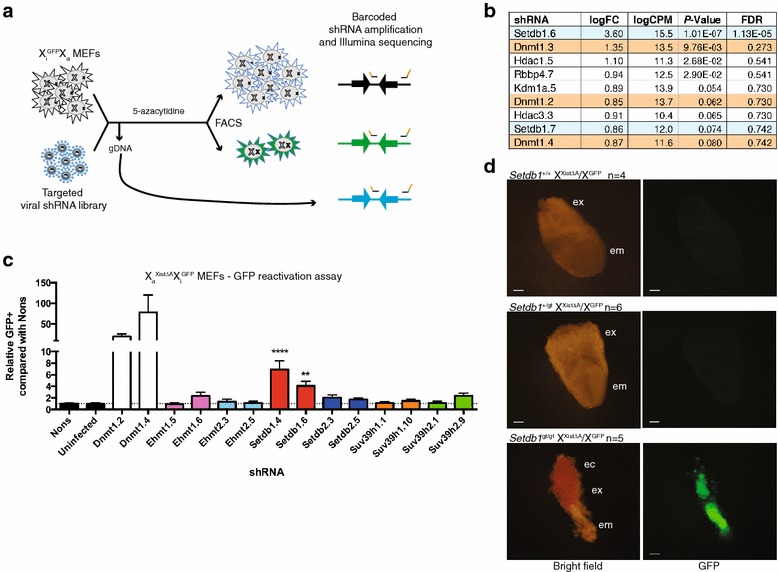
An in vitro screen for H3K9 methyltransferases involved in the maintenance of XCI identifies Setdb1. **a** MEFs were transduced with a pool of shRNA retroviruses and a DNA sample taken before 5-aza treatment (Additional file 3: Figure S3). After treatment, DNA was extracted from the GFP^+^ and GFP^−^ populations obtained by FACS. Hairpin sequences were amplified by PCR with a common forward primer and barcoded reverse primers. **b** The most significantly differentially represented hairpins (*p* < 0.1) with a positive fold change in the GFP^+^ sample compared to the GFP^−^ sample. *Dnmt1* positive control hairpins are highlighted in orange and *Setdb1* hairpins in blue. **c** Immortalised X_a_^XistΔA^X_i_^GFP^ MEFs were transduced with validated H3K9 methyltransferase shRNAs, treated with 5-aza and GFP reactivation assessed by FACS as shown in Figure S1B. n = 4, mean + s.e.m, one-way ANOVA with Dunnett correction for multiple testing, and only H3K9 methyltransferase shRNAs were included in the comparison. **p < 0.01; ****p < 0.001. **d** Bright field and GFP fluorescence images of representative E7.5 *Setdb1* ^+/+^, *Setdb1* ^+/gt^, and *Setdb1* ^gt/gt^ X_a_^XistΔA^X_i_^GFP^ embryos at 63 × magnification. Embryonic (em), extra-embryonic (ex) and ectoplacental cone (ec) tissues are indicated. Scale bar represents 200 µm. See also Additional files 3: Figure S3, 5: Figure S4, 4: Table S1, 6: Figure S5 and 7: Figure S6

Given our finding of H3K9 methylation enriched on the Xi, we were interested to test which methyltransferase was responsible for this methylation. Setdb1 has recently been identified as a H3K9 methyltransferase required for maintenance of silencing of a reporter allele knocked into the X-linked *Hprt* locus in mouse embryonic fibroblasts (MEFs) [20]. Concurrently, we also sought to identify H3K9 methyltransferases important for the maintenance of XCI. We used an X-linked GFP transgene that is subject to XCI (X^GFP^) [27] in MEFs. By natural immortalisation, we derived an X^GFP^X cell line with no expression of GFP due to the GFP transgene being on the Xi (X_a_X_i_^GFP^), which we validated using a combination of hairpins against genes known to be required for maintenance of XCI and two different treatment regimes of either a high or low dose of the DNA methyltransferase chemical inhibitor 5-azacytidine (5-aza) [14, 15] (Additional file 3: Figure S3). The high 5-aza treatment was used to provide high reactivation but low signal-to-noise, while the low-dose treatment provided low background but also low signal. Utilising these MEFs and high 5-aza, we performed a targeted pooled shRNA screen to identify genes involved in maintenance of XCI, with a pool of hairpins designed against known H3K9 methyltransferases, other genes required for XCI, gene silencing more generally and controls (Additional file 4: Table S1). Using this approach (Fig. 2a) and previously described bioinformatic analyses [28], we confirmed Setdb1 is required for maintenance of XCI (Fig. 2b, shRNA Setdb1.6, FDR 1.13 × 10^−5^). No shRNAs targeting other H3K9 methyltransferases gave a positive and significant readout.

To confirm the screen data, we used validated shRNAs (Additional file 5: Figure S4) against all six characterised H3K9 methyltransferases in individual X^GFP^ reactivation assays. We employed an X^Xist∆A^/X^GFP^ MEF cell line immortalised retrovirally via p53 knockdown, with low-dose 5-aza. As with our pooled screen, the only H3K9 methyltransferase that gave statistically significant reactivation of the X-linked GFP transgene upon depletion was Setdb1 (*p* < 0.0001, Fig. 2c). The level of reactivation was lower than the Dnmt1 knockdown positive controls, likely due to the weaker synergism between Setdb1 depletion and low-dose 5-aza than with Dnmt1 depletion, which was not apparent with high-dose 5-aza used in the pooled screen. These data suggest Setdb1 is the most dose-dependent H3K9 methyltransferase required for maintenance of XCI.

### Setdb1 is required for XCI in vivo

Minkovsky et al. identified a role for Setdb1 in maintenance of XCI in vitro [20], as we confirm above; however, the role of Setdb1 was unknown in vivo. To determine whether Setdb1 is required for XCI in vivo, we generated a *Setdb1* gene trap line of mice. The insertion site of the gene trap cassette is in intron 4 of *Setdb1* and the resultant allele (*Setdb1*^gt^) is predicted to produce a protein without functional domains (Additional file 6: Figure S5a, b). We confirmed reduced Setdb1 protein in *Setdb1*^+/gt^ MEFs by Western blot (Additional file 6: Figure S5c). Furthermore, this allele was homozygous lethal in males and females around E7.5 (Additional file 7: Figure S6; Fig. 2d), mimicking the knockouts for *Setdb1* [29] and suggesting the gene trap yields a null allele. By using the *Setdb1*^gt^ allele in combination with the *Xist*^∆A^ allele *in trans* to the X^GFP^ transgene, we found that at E7.5, *Setdb1*^gt/gt^ X_a_^Xist∆A^/X_i_^GFP^ embryos show GFP expression in both the embryo proper and the extra-embryonic tissues, whereas *Setdb1* wild-type controls show no GFP expression, as expected (Fig. 2d). In mice, cells of extra-embryonic tissues exhibit paternally imprinted X inactivation. Therefore, these data indicate Setdb1 is involved in imprinted XCI in vivo. However, it is difficult to decisively determine whether the GFP expression in the embryo represents a role for Setdb1 in random XCI in vivo, as the embryo is severely developmentally delayed, and at earlier stages of embryonic development random XCI has not yet occurred. We address the role of Setdb1 in earlier stages of XCI using differentiating embryonic stem cells in a later section (Fig. 5).

### ChIP-seq reveals Setdb1 is located on the inactive X

While Setdb1 depletion destabilises inactivation of X-linked reporter alleles, Setdb1 ChIP-seq has only been performed previously in male cells [30, 31]. To test whether Setdb1 has a direct effect on X inactivation, we performed ChIP-seq for Setdb1 in female MEFs. We used *Setdb1*^+/gt^*Xist*^∆A/+^*castaneus*/*domesticus* F1 hybrid MEFs, that we also treated with *Setdb1* knockdown to achieve the lowest possible levels of Setdb1 (Additional file 6: Figure S5d) (referred to subsequently as Setdb1 depleted). We compared these to female *Setdb1*^+/+^*Xist*^∆A/+^*castaneus*/*domesticus* F1 hybrid MEFs treated with non-silencing (Nons) shRNA (referred to as female) and male *Setdb1*^+/+^ X/Y *castaneus*/*domesticus* MEFs also with Nons shRNA (referred to as male).

**Fig. 3:**
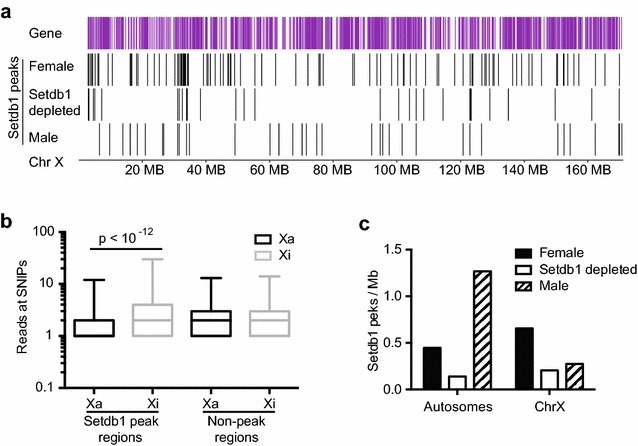
Setdb1 is enriched on the inactive X chromosome. Setdb1 ChIP-seq data from 129/CAST F1 MEFs: *Setdb1* ^+/+^ *Xist* ^∆A/+^ transduced with Nons (female) (n = 3) or *Setdb1* ^+/gt^ *Xist* ^∆A/+^ transduced with shSetdb1.6 (Setdb1 depleted) (*n* = 2) MEFs and Male *Setdb1* ^+/+^ X/Y MEFs transduced with Nons (male) (*n* = 3). The *Xist* ^∆A^ allele ensures the CAST X chromosome is the Xi. **a** Location of genes shown in *purple*, MACS2 called peaks of Setdb1 shown as *black lines* for each sample type along the X chromosome. **b** *Boxplot* showing the number of reads at informative SNPs from the Xi vs Xa, at Setdb1 peak and non-peak regions. *p* values shown were determined by Student’s two-tailed paired *t* test. **c** The number of Setdb1 peaks per Mb of the X chromosome or all autosomes is given for each of the sample types. Also see Additional file 8: Figure S7

We found there are more Setdb1 peaks on the female X chromosome than the male (Fig. 3a, c), consistent with a preference for the Xi over the Xa. These Setdb1 peaks are, as expected, quite narrow (approximately 300 bp). Therefore, when an allele-specific analysis was performed as described above, few peaks had informative SNPs; however, using those that are informative, there is on average a twofold enrichment for reads mapping to the Xi compared with the Xa at Setdb1 peak regions, but no difference at non-peak regions (Fig. 3b). Upon Setdb1 depletion, fewer Setdb1 peaks are observed on the X in females (Fig. 3c). The remaining peaks are smaller in amplitude (Additional file 8: Figure S7), but still spread along the length of the chromosome (Fig. 3a).

In addition to the differences in Setdb1 binding on the X between males and females, we also observed striking differences in Setdb1 localisation between the sexes, suggesting sex-specific roles for the protein. Setdb1 is more prevalent on Male autosomes than Female, whereas it is more prevalent on the female X than the male (Fig. 3a, c). Potentially the Xi acts as a sink for Setdb1, leaving less available to bind female autosomes. Upon Setdb1 depletion, there was global loss of Setdb1 peaks that was roughly equivalent on all chromosomes (Additional file 8: Figure S7).

### Setdb1 depletion alters the epigenetic state of the Xi

**Fig. 4:**
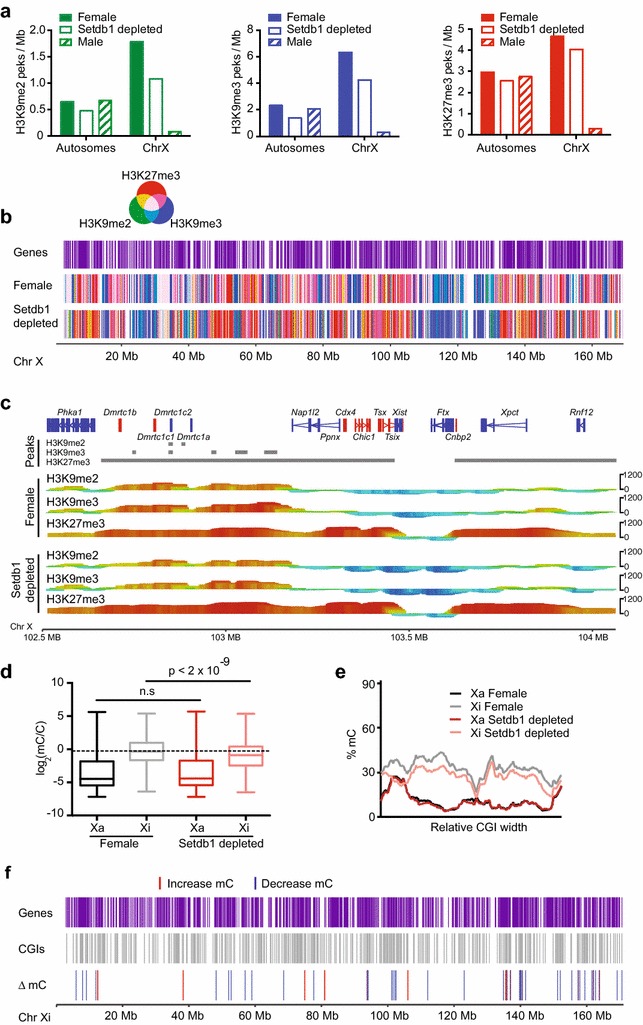
Setdb1 depletion results in losses of H3K9me2, H3K9me3 and DNA methylation spread along the inactive X chromosome. **a**–**c** ChIP-seq data from 129/CAST F1 MEFs: *Setdb1* ^+/+^ *Xist* ^∆A/+^ transduced with Nons (female) (*n* = 3) or *Setdb1* ^+/gt^ *Xist* ^∆A/+^ transduced with shSetdb1.6 (Setdb1 depleted) (*n* = 2) MEFs and Male *Setdb1* ^+/+^ X/Y MEFs transduced with Nons (male) (*n* = 3). The female and male samples are as shown in Fig. 1. **a** Peaks per Mb of chromosome for H3K9me2, H3K9me3 and H3K27me3, X chromosome shown separately to the X chromosome. **b** Location of genes (*purple*), peaks of H3K27me3 (*red*, n = 3), H3K9me2 (*green* n = 3), H3K9me3 (*blue* n = 3), as a merge of all three histone marks in female or Setdb1 depleted MEFs, as determined by ChIP-seq, derived from a Seqmonk browser track. **c** H3K9me2, H3K9me3 and H3K27me3 shown for the X inactivation centre (XIC). The genes within the XIC are shown, and beneath peaks called by Seqmonk in-built MACS peak caller as *grey bars*. Seqmonk tracks are also shown, where the height of the track indicates the read number related to H3. Significance of enrichment or depletion is given by the strength of colour—*red* is most enriched, *blue* is most depleted. **d**–**f** eRRBS data from the same samples as **a**–**c**. **d** Depletion of mC for individual CpGs on the Xa and Xi in Female (*n* = 3) and Setdb1 depleted female samples (*n* = 2), for CpGs within CGIs on the X chromosome (Student’s two-tailed *t* test) as log_2_(mC/C). *Dotted line* indicates median for Xi in Female. **e** Average %mC along the width of a CGI after normalising for CGI length for CpGs falling within CGIs on the X only. **f** Location on the Xi of the CpGs with significantly altered mC levels, with genes (*purple*), CGIs (*grey*), CpGs gaining mC (*red*) and CpGs losing mC (*blue*) upon Setdb1 depletion indicated, derived from a Seqmonk browser track. See also Additional files 10: Figure S9, 11: Figure S10

In order to establish the epigenetic consequence of Setdb1 depletion, we performed ChIP-seq for H3K9me2, H3K9me3, H3K27me3 in the afore-mentioned Setdb1-depleted female MEFs alongside controls. We additionally measured DNA methylation by enhanced reduced representation bisulphite sequencing (eRRBS) [32]. We found a roughly proportional loss of H3K9me2 and H3K9me3 on all female chromosomes upon depletion of Setdb1 in MEFs (Fig. 4a). Surprisingly, we also found a small loss of H3K27me3 on all chromosomes upon Setdb1 depletion (Fig. 4a), possibly due to a secondary effect of destabilised heterochromatin from H3K9me2/3 loss, or the recently reported functional interplay between Setdb1 and PRC2 [33]. Consistent with these data, we also observed a subtle reduction in H3K27me3 and H3K9me3 by Western blot in *Setdb1*^+/gt^ MEFs (Additional file 6: Figure S5c). Repressive histone marks are lost along the length of the X chromosome upon Setdb1 depletion (Additional file 9: Figure S8a, b), suggesting that Setdb1 depletion is not site specific but rather affects the whole Xi. However, it is interesting to note that despite these changes, the X chromosome remains largely covered in repressive histone marks (Fig. 4b).

One specific region of the X that is of high interest for X inactivation studies is the X inactivation centre (XIC): a 1.5-Mb region that encompasses *Xist* and many of the genes or DNA elements that control *Xist* expression. We found no differences in this region between Setdb1-depleted female samples and control female samples. All three marks can be found in the XIC, but H3K27me3 is most prominent, marking the majority of the XIC, leaving only *Xist* and the upstream region encompassing most of *Ftx* devoid of this repressive mark (Fig. 4c). These results are consistent with a previous report looking at a narrower region surrounding *Xist* by ChIP-chip for H3K27me3 and H3K9me2 [34].

While H3K9 methylation has been linked to DNA methylation on the Xi [20], no assessment has been made of the degree to which Setdb1 depletion alters DNA methylation on this chromosome. Using eRRBS in our MEFs system, as expected, we found dramatically higher levels of mC on the Xi compared to the Xa (Fig. 4d). In line with the loss of H3K9 methylation, we found a small but significant reduction in mC levels at CpG islands (CGIs) on the female Xi upon Setdb1 depletion (Fig. 4d). No effect was observed on mC at CGIs on the Xa (e.g. those associated with the *Rhox* cluster genes that are developmentally silenced, independent of XCI) or CpGs outside of CGIs on the Xi (Additional file 10: Figure S9a). We found similar results on autosomes: no effect at CpGs within or outside of CGIs (Additional file 10: Figure S9b, c). Interestingly, we observe a slight but significant decrease in mC at methylated CGIs (>20 % mC, Additional file 10: Figure S9d) suggesting a specific requirement for Setdb1 at CpGs with functional relevance for silencing. By profiling all CpGs within each CGI on the Xi we found mC loss was broadly uniform across the length of CGIs (Fig. 4e). Consistent with this, 18 CGIs showed significantly reduced mC, when all CpGs within the CGI are considered (Additional file 11: Figure S10). CGIs with depleted methylation distributed across the Xi, but because CGIs are typically at gene promoters, they were unsurprisingly located in gene-rich regions where H3K27me3, rather than H3K9me2/3, is abundant (Fig. 4f).

### Reactivation of X-linked genes upon depletion of Setdb1-mediated H3K9 methylation in MEFs is minor

As depletion of Setdb1 results in loss of repressive marks on the Xi (Fig. 4) and reactivates an X^GFP^ reporter (Fig. 2), we tested whether endogenous X-linked genes could also be reactivated by Setdb1 knockdown. By crossing *Mus musculus domesticus* 129/C57 *Setdb1*^+/gt^*Xist*^∆A/+^ dams with *Setdb1*^+*/*+^ PGK-X sires [34], which allow for allelic distinction due to a polymorphic X chromosome, we derived *X*^*Xist∆A*^*X*^*PGK*^*Setdb1*^+*/gt*^ and *X*^*Xist∆A*^*X*^*PGK*^*Setdb1*^+*/*+^ female MEFs. Again *Setdb1*^+*/gt*^ cells were transduced with Setdb1 hairpins and wild-type cells with Nons, then subjected to RNA-seq analysis. We first confirmed that only the PGK strain wild-type allele of *Xist* was detectably expressed (Additional file 12: Figure S11a). We found depletion of Setbd1, even in combination with inhibition of DNA methylation via 5-aza treatment, had only a very minor ability to reactivate endogenous X-linked genes (Additional file 13: Table S2), likely reflecting redundancy in modes of maintaining silencing on the Xi. Moreover, these data suggest that the X-GFP transgene used for Fig. 1 is more labile than endogenous genes, probably because it exists as a multi-copy array, and Setdb1 is known to silence repetitive transgenes [35].

### Depletion of Setdb1-mediated H3K9 methylation during establishment of XCI results in failure to silence endogenous X-linked genes

**Fig. 5:**
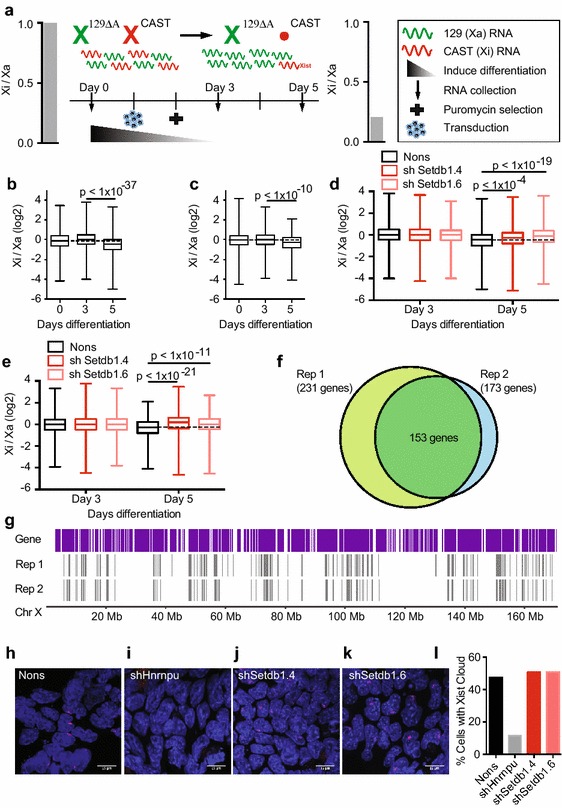
Setdb1-mediated H3K9 methylation is required for silencing at hundreds of genes spread along the inactive X chromosome. **a** Schematic representation of these RNA-seq experiments. Female *Xist* ^∆A/+^ 129/CAST ES cells, where the *Xist* ^*∆A*^ allele forces the CAST-derived X to be the obligate Xi, were differentiated by transitioning them from 100 % 2i plus LIF media into differentiation media in 4 increments over 4 days (see “Methods” section for full details). RNA was collected on day 0 and differentiation induced. Cells were transduced the following day with shSetdb1.6 or Nons control, puromycin selection for transduced cells was performed the following day, then RNA collected at day 3 and day 5 post-differentiation induction. In this system, XCI can be observed as a decrease in exonic reads mapping across SNPs to the CAST X compared to the 129 X; i.e. a decrease in the Xi/Xa ratio. **b**, **c** *Box plots* showing the Xi/Xa ratio over the differentiation time course for the Nons sample, suggesting that XCI is occurring as expected in both replicates 1 (**b**, *n* = 1221–1769 SNPs) and 2 (**c**, *n* = 874–1649 SNPs). **d**, **e** The Xi/Xa ratio is plotted for replicates 1 (**d**, *n* = 1033–1808 SNPs) and 2 (**e**, *n* = 874–1696 SNPs) at day 3 and day 5 of differentiation for cells transduced with shSetdb1.4, shSetdb1.6 or Nons. For **b**–**e**, *p* values are shown (Student’s two-tailed *t* test). **f** Venn diagram showing genes containing at least one reactivating SNP in replicates 1 and 2. Reactivating SNPs are defined as having a higher Xi/Xa ratio in both the shSetdb1.4 and shSetdb1.6 samples compared to the Nons. **g** Location on the Xi of genes carrying reactivating SNPs, derived from a Seqmonk browser track (www.bioinformatics.babraham.ac.uk/projects/seqmonk/). **h**–**l** RNA FISH stained for *Xist* (*red*) and DAPI (*blue*) in freshly derived day 5 differentiated female Bl/6 ES cells, following treatment with a negative control Nons (**h**), positive control shHnrnpu (**i**), shSetdb1.4 (**j**) or shSetdb1.6 (**k**). **l** Quantification of *Xist* clouds observed by FISH. See also Additional files 12: Figure S11, 14: Figure S12

In order to subvert some of the redundancy involved in the maintenance of XCI, we tested the role of Setdb1-mediated H3K9 methylation by depleting Setdb1 in differentiating ES cells, when X inactivation is first being established. We produced female ES cells from *Mus musculus castaneus* sires mated with *Mus musculusdomesticus* 129 *Xist*^*∆A/*+^ dams. A resultant *Xist*^∆A/+^ 129/CAST F1 line of female ES cells was used for allele-specific RNA-seq, with and without *Setdb1* knockdown, throughout ES cell differentiation. The cells were transduced on the day differentiation was induced to avoid the premature differentiation upon Setdb1 depletion shown previously [30, 31, 36, 37]. RNA was sampled throughout differentiation, for two independent experiments, and subjected to next-generation sequencing (NGS) (Fig. 5a). A third replicate proved uninformative as analysis of X chromosome content showed the cells had become XO (data not shown).

As expected, we observed the expression of pluripotency genes in the undifferentiated ES cells that were rapidly silenced upon differentiation, while differentiation markers were activated (Additional file 14: Figure S12). *Xist* expression was detected 2 days following complete withdrawal of pluripotency media (day 5 in our differentiation system) and was only ever detectably expressed from the wild-type castaneus allele (Additional file 12: Figure S11b). Importantly, Setdb1 knockdown was maintained throughout differentiation, but no other H3K9 methyltransferases were affected (Additional file 14: Figure S12). Indeed, no genes reached genome-wide significance for differential expression upon Setbd1 knockdown, which is supportive of Setdb1 playing a direct role in XCI. By analysing the ratio of *castaneus* to *domesticus* reads (Xi/Xa) at all X-linked genes, we observed XCI during differentiation in both replicates (Fig. 5b, c). For both replicates, *Setdb1* knockdown samples displayed failure of silencing at day 5 that was not observed at previous time points, that we observe both by analysing the Xi/Xa ratio (Fig. 5d, e) and by looking at the average level of expression from the putative Xi (Additional file 12: Figure S11c, d). The true magnitude of this effect is likely muted in our system by the presence of XO cells. Failure of silencing is observed at 231 and 173 genes for replicates 1 and 2, respectively, with an overlap of 153 genes (~14.4 % of SNP containing X-linked genes) (Fig. 5f, Additional file 15: Table S3). For genes with multiple SNPs, the majority of SNPs within each gene are in agreement. These SNPs are spread along the length of the chromosome (Fig. 5g), demonstrating Setdb1-mediated H3K9 methylation is required for silencing at scores of X-linked genes during early ES cell differentiation.

Interestingly, despite the consistency of genes that fail to silence between replicates 1 and 2, these genes did not necessarily correlate with sites that lose Setdb1 binding, histone methylation or DNA methylation. These data raise the possibility that loss of silencing is due to failure to appropriately establish or maintain the chromosome conformational state required for silencing. Given the role of H3K9 methylation in silencing repetitive elements [25] and the known role of silencing of young LINE1 repeats in creation of a silent nuclear compartment in the early stages of X inactivation, i.e. a specific chromosome conformation required for silencing [38, 39], we assessed X chromosome-specific expression of the different repeat classes in our RNA-seq data set from early ES cell differentiation. We found only the LTR class of X-linked repeats were significantly overrepresented among differentially expressed mappable repeats upon Setdb1 depletion (*p* < 1.68^−05^ Chi-squared test, Additional file 16: Table S4). The requirement for Setdb1 at LTRs has been noted before [40]; however, consistent with our observations that Setdb1 depletion has a more pronounced effect on the X chromosome, we find no significantly variable repeat classes when repeats on all chromosomes are considered.

One possible explanation for this failure to silence many genes on the inactive X could be destabilisation of *Xist* expression; however, our RNA-seq analysis of early differentiating ES cells shows no significant change in *Xist* expression after Setdb1 knockdown (Additional file 14: Figure S12). To complement the RNA-seq, we assessed the ability of *Xist* to form its distinctive “cloud” at the Xi by RNA FISH at day 5 of ES cell differentiation and found no difference between Setdb1 knockdown and Nons control, whereas knockdown of positive control Hnrnpu which is required for *Xist* RNA coating [41] reliably destabilised the *Xist* RNA cloud (Fig. 5h–l). Consistent with this, we observed no changes to the chromatin state at the XIC upon Setdb1 depletion in MEFs (Fig. 4c). These data suggest depletion of Setdb1-mediated H3K9 methylation does not alter *Xist* expression, but is instead required at the late establishment or early maintenance phase of XCI.

### Setdb1-mediated H3K9 methylation is required for silencing at autosomal genes

**Fig. 6:**
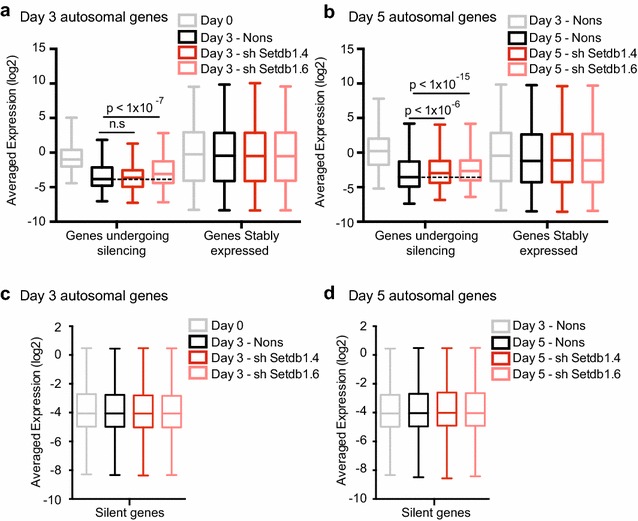
Setdb1-mediated H3K9 methylation is also required for autosomal gene silencing. RNA-seq data from differentiating *Xist* ^∆A/+^ 129/CAST ES cells transduced with the indicated shRNAs (as described in Fig. 5). **a**, **b** Data from both replicate experiments showing the average expression of all autosomal genes proximal to Setdb1 ChIP-seq peaks, that either become silenced, or maintain expression from day 0 to day 3 (**a**, *n* = 71 and 3487 genes, respectively) and day 3 to day 5 (**b**, *n* = 171 and 3487 genes, respectively). These are genes that are either establishing silencing or being stably expressed. **c**, **d** Data from both replicate experiments showing the average expression of all autosomal genes (*n* = 2224) that remain silent in the Nons samples from day 0 to day 3 (**c**) and day 3 to day 5 (**d**). These are genes that are maintaining silencing. For a-d, p-values were determined by Student’s two-tailed *t* test

We next sought to understand whether the greater role we observed for Setdb1-mediated H3K9 methylation while silencing is being established, compared with when it is just being maintained, was a peculiarity of the Xi or if this applied more broadly at autosomal loci. We categorised all genes within close proximity to Setdb1 ChIP-seq peaks from our MEF data set (Fig. 2) as being progressively silenced or maintaining stable levels of detectable expression during ES cell differentiation. Upon Setdb1 knockdown, there was significant failure of repression for the cohort of autosomal genes that are undergoing silencing, for one Setdb1 hairpin at differentiation days 3 and for both Setdb1 hairpins at day 5 (Fig. 6a, b), just as we found for X-linked genes. By contrast, autosomal genes that maintain expression showed no change. Finally, we looked at autosomal genes that maintain their silent state throughout ES cell differentiation and, similarly to what we saw for XCI in MEFs, could detect no reactivation of silent genes upon depletion of Setdb1, both at days 3 and 5 (Fig. 6c, d). These data suggest that Setdb1-mediated H3K9 methylation is broadly required for the late stages of establishment or earliest stages of maintenance of gene silencing, but has only a minor role in maintaining silencing once it is already set up, both on the Xi and at autosomal genes.

## Discussion

Over the past 15 years, it has been contentious whether H3K9 methylation is enriched on the mouse inactive X, and even in species where H3K9 methylation has been accepted to accumulate on the Xi, its role has remained unclear. Here we profiled the repressive histone marks H3K9me2, H3K9me3 and H3K27me3 on the mouse Xi. A striking feature of these profiles is the banded pattern they exhibit, with H3K27me3 marking gene dense regions and H3K9 methylation being predominant at gene poor regions, thereby creating distinct interspersed banding patterns along the chromosome and further enhancing our view of just how densely covered the Xi is with repressive histone modifications. Immunostaining of metaphase spreads has shown non-overlapping domains of facultative heterochromatin on the Xi defined by either H3K9me3 or H3K27me3 for human [24], cow [42] and vole [43]. More recently in mouse, H3K27me3 localisation was found to correlate with genes [22], but ours is the first description of discrete H3K9 methylated domains in mouse and indeed the first report of H3K9me3 on the murine Xi. Previous reports found no enrichment of H3K9me3 on the Xi by immunofluorescence [19]. Our data are consistent with these earlier findings, as we don’t find enrichment for H3K9me3 on the Xi compared with autosomes meaning enrichment on the Xi would be difficult to discern by immunofluorescence. Ours is also the first ChIP-seq analysis of H3K9me2 on the mouse inactive X. While early immunofluorescence studies identified H3K9me2 on the Xi, this work was later called into question due to antibody cross-reactivity with H3K27me3 [6]. Unfortunately, the paucity of SNPs at H3K9me2 peak regions on the X does not allow us to make a conclusion about the presence of this mark on the Xi; however, our comparison with the male X chromosome would suggest that it is. This combined with recent immunostaining evidence showing dense H3K9me2 regions colocalising with the dense H3K27me3 of the Xi [20], our own immunofluorescence study and an older study using an antibody against H3K9me2 without cross-reactivity issues [26], suggest further study of H3K9me2 on the Xi is warranted.

To functionally discern the role of H3K9 methylation on the Xi, we performed paired pooled and individual shRNA screens to identify which of the seven known H3K9 methyltransferases were involved in maintenance of XCI, using an X-linked GFP transgene. This approach revealed Setdb1 to be the most dose-dependent H3K9 methyltransferase for maintenance of XCI. Several other studies have investigated H3K9 methyltransferases in maintenance of XCI, including Ehmt2, Suv39h1 and Suv39h2 [20, 44, 45]. Consistent with our findings, only Setdb1 has been shown to play a role in XCI [20]. We additionally found no role for the remaining untested H3K9 methyltransferases Ehmt1 and Setdb2. As we performed our experiments by knockdown rather than knockout, we cannot exclude that the other H3K9 methyltransferases remain functional at depleted doses or that there is some compensation between various H3K9 methyltransferases such as those that are most closely related, e.g. Ehmt1 and Ehmt2. However, Setdb1 is clearly the most dose-sensitive H3K9 methyltransferase required for maintenance of XCI. Interestingly, while H3K9 methyltransferases are known to act as a multi-meric complex in some instances [46], here we find depletion of Setdb1 is sufficient to destabilise XCI. This is similar to what has been reported for silencing of LTR repeats by Setdb1 [40].

Using Setdb1 depletion as a tool to analyse the role of H3K9 methylation on the Xi, we analysed the epigenetic and transcriptional consequence of Setdb1 depletion in MEFs, where XCI is already completely established. Upon Setdb1 depletion, we observed a significant reduction in H3K9 methylation on the X; however, regions that lose methylation were often also marked by H3K27me3 or at the periphery of domains, meaning the distinct interspersed pattern of H3K9 and H3K27 methylation remained largely intact (Fig. 4c). This suggests these domains are stable once silencing is established, possibly protected by other H3K9 methyltransferases acting redundantly. Interestingly, a previous report identified the chromodomain protein Cdyl on the Xi [44]. They found that Cdyl’s recruitment to chromatin was dependent on both H3K9me2 and H3K27me3 and that it binds the H3K9 methyltransferase Ehmt2 (G9a), which is also enriched on the Xi. In combination with their data, our data suggest Setdb1 may establish H3K9 methylation on the Xi, providing a binding site for Cdyl, which could be involved in its maintenance through recruitment of Ehmt2 or other H3K9 methyltransferases such as Setdb1. However, there is likely redundancy between Ehmt2 and Setdb1, given that Ehmt2 has not been found to alter maintenance of the silent state of X-linked reporters here, or by others [20, 45], that Setdb1 itself has limited effects on the maintenance of endogenous X-linked and autosomal gene silencing, and that Setdb1 depletion does not completely ablate H3K9 methylation on the Xi. Likely this explains why upon Setdb1 depletion in MEFS we observed only a subtle effect on maintenance of X-linked gene silencing.

In addition to changes in H3K9 methylation upon Setdb1 depletion, we observed a decrease in DNA methylation at a subset of CpG islands on the Xi. This is consistent with the interplay between H3K9 methyltransferases, H3K9 methylation and DNA methyltransferases [47–50], and the synergism between Setdb1 and methyl binding domain protein 1 (Mbd1) that binds methylated CpGs for the maintenance of XCI [20]. Interestingly, depletion of Dnmt1 disrupts only the maintenance of random XCI, which was shown by normal expression of an X-linked reporter allele at E8.5 in both the embryo and placenta, but reactivation at E9.5 exclusively in the embryo [14]. This is in contrast to what we observed for Setdb1: firstly, we observed an effect for imprinted X inactivation; secondly, we observed an effect at E7.5. At this embryonic stage, cells are heterogeneously establishing or maintaining X inactivation, therefore raising the possibility that Setdb1-mediated H3K9 methylation could be involved in both stages of silencing. The earlier timing also places H3K9 methylation as a critical event in XCI that must occur prior to DNA methylation.

While we saw a striking effect on expression of the X-GFP transgene in Setdb1 null embryos, we saw only a very small reactivation of the same transgene in MEFs and saw even lower reactivation of endogenous X-linked genes in the same cells upon Setdb1 depletion. There are many possible contributors to these observations: knockout rather than knockdown of Setdb1; functional redundancy in maintenance of XCI and a requirement for Setdb1 at earlier stages of XCI. To address the latter two points and broaden our understanding of the role of Setdb1-mediated H3K9 methylation in inactivation of endogenous X-linked genes, we analysed the effect of depleting Setdb1 while XCI is first being set up in differentiating female ES cells. Using allele-specific RNA-seq, we show Setdb1-mediated H3K9 methylation has a striking role at this time, both on the Xi and at autosomal loci genome-wide. In each case hundreds of genes fail to become appropriately silenced, representing a central role for H3K9 methylation in setting up their silent state. Importantly, Setdb1-mediated H3K9 methylation has a larger effect when depleted while silencing is being established, than when it has already been completed and is only being maintained, both on the Xi and at autosomal genes. The functional role of Setdb1-mediated H3K9 methylation is in contrast to the role of PRC2-mediated H3K27me3. H3K27me3 is not required to establish or maintain random XCI, as determined by knockout of the PRC2 member Eed [12], nor is it sufficient to elicit gene silencing following expression of a silencing defective *Xist* [7, 51].

## Conclusions

Here we have found that H3K9 methylation is enriched on the inactive X chromosome, particularly at intergenic, repetitive regions. We identified Setdb1 as the most dose-dependent H3K9 methyltransferase involved in the maintenance of X inactivation. In analysing the X-linked genes that fail to become silenced upon Setdb1 depletion in differentiating female ES cells, we found that they are spread along the X chromosome. Interestingly, these genes do not necessarily correlate with sites that lose Setdb1 binding, H3K9 methylation or DNA methylation upon Setdb1 knockdown in MEFs. Moreover, we observe differential expression of X-linked LTR repeats upon Setdb1 depletion. These data raise the possibility that H3K9 methylation plays a role in both the late establishment and early maintenance of silencing. Furthermore, this gene silencing may be facilitated via silencing of repeats, which alter chromosome conformation. This possibility is something of high interest for us to investigate in the future.

## Methods

### Animal strains and husbandry

All animals were kept and treated under Walter and Eliza Hall Institute Animal Ethics Committee approved protocols. D4/XEGFP were obtained from Jackson labs and backcrossed onto the C57BL/6 background. Xist^∆A^ mice [21] were obtained from Dr Graham Kay, Queensland Institute of Medical Research, and kept on a 129 background. Setdb1 gene trap ES cells (Bay Genomics RRK370) were used to derive Setdb1 gene trap mice, which were backcrossed for > 10 generations onto the C57BL/6 background. Oligonucleotides used for genotyping are given in Additional file 17: Table S5.

### Derivation and culture of MEFs

MEFs were obtained from E13.5 embryos. Cells were cultured in DMEM with 10 % (v/v) FBS at 37°C and 10 % (v/v) CO_2_. To immortalise primary MEFs, cells were cultured for >20 passages or transduced with LMH-p53 retrovirus and selected with 300 µg/mL hygromycin for 7 days.

### Production of retrovirus and transduction

Retrovirus was produced as described [52]. MEFs were transduced with retrovirus in medium containing 4 μg/mL polybrene, and cells were selected with 3–5 µg/mL puromycin after 24 h.

### qRT-PCR

Knockdown efficiency of shRNA retroviral constructs was determined using Roche Universal Probe Library (UPL) assays. Relative mRNA expression levels were determined using the 2^–ddCt^ method, with *Hmbs* and/ or *Hprt* as house-keeping controls. Probe numbers and oligonucleotide sequences appear in Additional file 17: Table S5.

### X reactivation assay

MEFs were transduced with shRNA retroviruses, selected with puromycin, then treated with 10 µM 5-azacytidine (Additional file 3: Figure S3). Cells were prepared in KDS-BSS with 2 % (v/v) FBS and a cell viability dye: either 0.5 μg/mL propidium iodide (PI), 16 µg/mL FluoroGold or 0.5 µg/mL LIVE/DEAD Fixable Far Red. Cells were analysed using a BD LSR II or LSRFortesssa cell analyser. Cells were prepared similarly for sorting using a FACSAria. Flow cytometry data were analysed using FlowJo.

### shRNA screen analysis

MEFs were transduced with a pool of shRNA retroviruses (Additional files 4: Table S1, 18: Table S6). An initial sample was taken, and the remaining cells were used in an X reactivation assay. After 5-azacytidine treatment, the GFP^+^ and GFP^−^ populations were purified by FACS.

Genomic DNA was extracted using a Qiagen DNeasy Blood and Tissue Kit. shRNA integrants were amplified using AmpliTaq Gold DNA polymerase with a common forward oligonucleotide and barcoded reverse oligonucleotide. A total of 168-bp PCR products were quantified using a Bioanalyser or Tapestation, pooled in equal quantities and purified by gel extraction using a QIAquick Gel Extraction Kit.

Illumina next-generation sequencing was performed using a HiSeq 2000. A total of 100-bp single-end reads were obtained at a cluster density of 130 million reads per lane. Due to the similarity of the barcodes used for hairpins amplification, only 60 % of each lane contained shRNA-sequencing samples. The remaining 40 % of the lane was taken up with control PhiX DNA or RNA sequencing libraries.

Data were analysed essentially as described [28], using edgeR [53, 54]. The hairpin and barcode sequences were extracted from each sequencing read and matched to known hairpins included in the screen and the barcodes used for the amplification of each sample. The number of reads for each hairpin was tallied for each sample. Hairpins with fewer than 1000 reads in any of the replicates at the initial time point were excluded. Exact testing [55] was applied to assess the differences in hairpin abundance between different sample groups.

### E7.5 embryos

Pregnant females were killed at E7.5 by CO_2_ asphyxiation. The uterus was dissected, and the deciduae were removed and washed in PBS. Embryos were dissected from the deciduae and transferred into PBS with 0.1 % (v/v) Tween 20. Bright field and GFP images were taken using an Olympus SZX16 fluorescence stereomicroscope. Embryos were transferred into DNA lysis solution, and DNA was obtained for genotyping.

### ES cell culture

Blastocysts and ES cells were cultured on gelatin-coated tissue culture ware at 37 °C in a humidified atmosphere with 5 % (v/v) carbon dioxide and 5 % (v/v) oxygen. To derive ES cells, blastocysts were flushed at E3.5 from superovulated pregnant females and cultured in 2i+LIF medium [56]. Inner cell mass outgrowths were trypsinised for 2 min, before they were mechanically disrupted in 2i+LIF and replated. Once large colonies had formed, they were passaged as ES cell lines.

### ES cell differentiation

ES cells that had been cultured in 2i+LIF medium were seeded into 75 % 2i+LIF medium 25 % ES cell DMEM + FBS. After 24 h, the medium was changed to 50 % 2i+LIF medium 50 % ES cell DMEM + FBS. After 48 h, the medium was changed to 25 % 2i+LIF medium 75 % ES cell DMEM + FBS. After 72 h, the cells were split and seeded at 10^4^ cells per cm^2^ in 100 % ES cell DMEM + FBS.

### ChIP-seq analysis

*Xist*^∆A/+^ 129/CAST MEF cells for ChIP-seq were cultured and transduced as above. ChIP-seq was performed as previously described [57], with some modifications to the shearing, immunoprecipitation and cross-link reversal methods. Chromatin was sheared with a Covaris S220 sonicator. Immunoprecipitation was performed overnight at 4 °C with 2 μg of either anti-Setdb1 (Santa Cruz Biotechnology, sc-66884, lot L1812), anti-H3 (Abcam, ab1791, lot GR135321-1), anti-H3K9me2 (Abcam, ab1220, lot GR139816-1), anti-H3K9me3 (Abcam, ab8898, lot GR130993-1) or anti-H3K27me3 (Millipore, 07-449, lot 2194165). Anti-H3K9me2 and anti-H3K9me3 antibodies have been known to suffer from cross-reactivity. The antibodies we employed were tested extensively by Abcam, using peptide competition followed by Western blot and ChIP-qPCR for known positive controls for these and other histone marks. Importantly, the anti-H3K9me2 has no detectable cross-reactivity with other histone marks; however, the anti-H3K9me3 has subtle cross-reactivity with H3K27me3, detectable by peptide competition. Our data show distinctly different patterns of H3K27me3 and H3K9me3 throughout the genome, for example on the X chromosome (Fig. 1c, Pearson correlation *r* = −0.25), suggesting this subtle cross-reactivity is unlikely to be relevant to our analyses.

Immunocomplexes were collected using Protein G Dynabeads (Life Technologies). Cross-links were reversed by heating at 65°C for 4 h, followed by RNaseI and proteinase K digest, and DNA was purified with the ChIP Clean and Concentrator Kit (Zymo). ChIP-seq libraries were created using the TruSeq DNA sample preparation kit (Illumina), and sequencing on the Illumina HiSeq 2000 platform (100 bp single-end) was performed by the Australian Genome Research Facility (AGRF, Melbourne).

Sequencing reads were aligned to a custom version of the mouse genome (mm10) where SNPs between *Mus musculus castaneus* and *Mus musculusdomesticus* were n-masked using VCF file obtained from the Mouse Genomes Project to eliminate mapping bias between alleles. Mapping was performed using Bowtie2 [58]. Sequencing data were analysed with the aid of the Seqmonk v0.29.0 software (www.bioinformatics.babraham.ac.uk/projects/seqmonk/). Samples were pooled in Seqmonk, and broad peaks were called for all histone samples using the MACS style caller within the Seqmonk package (settings for 10 kb fragments, *p* < 1 × 10^−05^) and the H3 samples as the control. For narrow Setdb1 peak calling, samples were pooled using samtools merge [59] and WCE samples used as the control for MACS2 [60] with down sampling and a *p* < 0.001. Chromosome lengths given in Seqmonk were used to determine ChIP peak per Mb. Meta-gene analysis of histone peaks was performed in Seqmonk by quantifying reads in 10 kb windows sliding by 5 kb. Data were produced using the Quantitation Trend Plot tool within Seqmonk and then normalised by subtracting the H3 values at each position. Seqmonk browser tracks were produced by quantifying regions over 50 kb bins, sliding by 5 kb each time, then normalised for library depth with Match normalisation, and the appropriate H3 sample subtracted for each genotype.

Enrichment analysis for genes, pseudogenes and histone peaks was performed by determining the percentage coverage over 50 kb bins along the female X chromosome. Gene and pseudogene locations were obtained from Seqmonk. Location of histone peaks was determined from our ChIP-seq data sets.

Genome browser tracks for chromatin marks were plotted using Gviz package (version 1.7.10) [61] in Bioconductor [62]. Reads corresponding to regions of interest were extracted from individual bam files and library-size normalised. Effective library sizes were calculated using csaw package based on a previously described approach [63]. Briefly, reads across the genome were counted in 10 kb bins for each library and the counts were used to compute normalisation factors using the TMM method [54]. Read depth was normalised by subtracting read coverage at corresponding positions in the H3 reference sample and plotted with 10 kb smoothing window.

### RNA-seq in MEFs

Primary X^*Xist*∆A^/X^PGK^ MEFs were transduced with retroviruses and selected with puromycin after 24 h. Three days after transduction, cells were cultured with or without 5-azacytidine for 24 h and harvested for RNA 7 days post-transduction and sequenced on an Illumina HiSeq 2000 for 100-bp single-end reads at the AGRF. Reads were aligned to the mm9 mouse reference genome (chr X only) using TopHat [64] with Bowtie1 [65]. Variants were identified in the PGK sample using samtools mpileup [59]. Reference and alternate allele counts for these variants were obtained using samtools pileup and aggregated across replicate samples. Variants were considered as PGK SNPs if the reference allele did not appear in the PGK sample and the alternate alleles did not predominate in the control sample. The allelic differences between control and treated samples were compared at the gene level by combining allele counts across all variants that occurred within the start and end position of each gene and applying Fisher’s exact test.

### RNA-seq in ES cells

For RNA-seq analysis *Xist*^∆A/+^ 129/CAST ES cells were differentiated as described above, with the difference that cells were transduced at the same time as differentiation was induced. Undifferentiated cells were maintained in 2i+LIF media, and RNA was collected as the cells were transferred to 75 % 2i+LIF medium plus 25 % ES cell DMEM + FBS (day 0 time point) and transduced with retrovirus containing shSetdb1.6 or Nons. Puromycin selection was performed the following day in 25 % 2i+LIF medium plus 75 % ES cell DMEM + FBS. RNA was then collected 1 (day 3 time point) and 3 (day 5 time point) days later. RNA was extracted using RNeasy columns (Qiagen). Sequencing libraries were prepared using the TruSeq RNA sample preparation kit (Illumina) and sequenced on the Illumina HiSeq 2000 platform (100 bp single-end) at the AGRF. Reads were mapped using the Subread [66] aligner to the mm10 genome, and gene-level counts were obtained using the featureCounts procedure and RPKM values (reads per kilobase per million) values calculated. Allele-specific counts were obtained using samtools pileup for SNPs between the *Mus musculus castaneus* and *Mus musculusdomesticus* strains on the X chromosome listed in the Mouse Genomes Project vcf file (referred to above). To determine the Xi/Xa ratio only SNPs within exons and covered by at least 10 reads were considered. To avoid dividing by zero, 1 was added to each count before the ratio of Xi/Xa was calculated. SNPs classed as reactivating were defined as having a larger Xi/Xa ratio for both shSetdb1.4 and shSetdb1.6 compared to Nons, in both of the replicate experiments. To determine absolute allele-specific expression, individual bam files for reads mapping to 129 and CAST were produced. These files were then analysed in Seqmonk using the RNA-seq quantitation pipeline and Match distribution quantitation.

For analysis of genome-wide expression, bowtie2 mapped bam files for replicates 1 and 2 were analysed within Seqmonk, using the RNA-seq quantitation pipeline and Match distribution quantitation, before being averaged. Genes were classified as being silenced if they had a greater than 2 log_2_ fold change between the Nons sample and the Nons sample at the previous time point, and additionally were within 200 kb of a Setdb1 peak. Genes with stable expression were defined as having less than 0.5 log_2_ fold change between Nons samples. Genes classified as maintaining silencing had an expression level below 0.5 in all Nons samples throughout the differentiation time course.

For expression analysis of repeat classes, either genome-wide or X chromosome-specific bam files were used as input for analyzeRepeats.pl, using the “repeat” argument and the mm10 reference genome [67]. Values for replicates 1 and 2 were averaged, and genes were classed as being differentially expressed if they had a log_2_ fold change greater than 1 between Setdb1 depleted and Nons samples. Repeat classes overrepresented in the differentially expressed group were determined by Chi-square test.

### eRRBS and analysis

eRRBS was performed by the Epigenomics Core Facility at Weill Cornell Medical College. Briefly, DNA was isolated from transduced *Xist*^∆A/+^ 129/CAST MEF cells using a DNeasy Blood and Tissue Kit (Qiagen) and shipped to Weill Cornell Medical College where libraries were produced using the eRRBS method [32]. Sequencing was performed on the HiSeq 2000 for single-end 100-bp read lengths using dark-cycle sequencing settings [68] to eliminate sequencing issues arising from low complexity introduced by the MspI cut site. Quality control was performed using FastQC (www.bioinformatics.babraham.ac.uk/projects/fastqc/). Sequencing reads were mapped to a bisulphite-converted version of the n-masked mm10 genome described above which was created using Bismark [69]. Adapter trimming was performed with TrimGalore (www.bioinformatics.babraham.ac.uk/projects/trim_galore/). Sequences were mapped to the bisulphite-converted n-masked mm10 genome using Bismark, before the 7 5’ most bases and 8 3’ most bases were trimmed based on m-Bias [61] results produced by Bismark. Reads were then split into either *Mus musculus castaneus* or *Mus musculusdomesticus* using SNPsplit (www.bioinformatics.babraham.ac.uk/projects/SNPsplit/) where C/T SNPs are excluded to eliminate errors due to the bisulphite conversion. Methylation calls were made using the Bismark Methylation Extractor [69] with further analysis performed using the Seqmonk software. Only CpG sites covered by more than 20 reads were considered for analysis. Our list of differentially methylated CpG sites was derived from CpGs that have a statistically significant change in percentage mC (Student’s two-tailed t-test) when the three replicate experiments were considered. Only reads mapping to the *Mus musculus castaneus* genome were considered and only for CpGs on the X chromosome that lie within CpG islands. Locations of CGIs were obtained from the Seqmonk package.

### Western blot

Cultured MEFs were washed with cold PBS buffer and then lysed with KALB lysis buffer (150 mM NaCl, 50 mM Tris-HCl pH 7.5, 1 % (v/v) Triton X-100, 1 mM EDTA pH 7.5) supplemented with 1 mM sodium vanadate, 1 mM PMSF and protease inhibitors (complete mixture tablets; Roche) on ice for 30 min. Insoluble material was removed by centrifugation at 15,000 g at 4°C for 5 min. Total protein concentration in the whole cell extract was quantified using the BCA protein assay kit (Pierce) following manufacturer’s instructions. Proteins were resolved by 4–12 % SDS-PAGE (Invitrogen), transferred to PVDF membranes (Osmonics; GE) and blocked with 5 % (w/v) skim milk powder in 0.1 % (v/v) Tween-PBS for 1 h at room temperature. Membrane was incubated overnight with anti-Setdb1 antibody (1:2000 diluted; Santa Cruz, sc-66884) and anti-Actin antibody (1:2000 diluted; Santa Cruz, sc-1616), anti-H3K27me3 antibody (1:2500; Millipore 07-449), anti-H3K9me2 antibody (a:1000; Abcam, ab1220), anti-H3K9me3 (1:1000; Millipore, 07-442) at 4 °C followed with horseradish peroxidase (HRP)-conjugated secondary antibodies. Membrane was visualised using ECL system (Immobilon; Millipore) following manufacturer’s instructions.

### Immunofluorescence

Immunofluorescence was performed as described [70], with modifications. The cells were mounted in Vectashield HardSet mounting medium with DAPI (Vector Laboratories). The primary antibodies used were H3K27me3 (1:100 dilution, Millipore 07-449), H3K9me2 (1:100 dilution, Abcam ab1220) and H3K9me3 (1:50, Abcam ab8898). The cells used were MEFs carrying a GFP knockin at the Smchd1 locus, enabling detection of Smchd1 protein. Secondary antibodies used were donkey anti-mouse IgG Alexa Fluor^®^555 conjugate (1:500, ThermoFisher A-31570) and goat anti-rabbit IgG Alexa Fluor^®^555 conjugate (1:500, ThermoFisher A-21428). Cells were visualised on an Elite Widefield microscope (DeltaVision). To determine overlap between Smchd1 and the histone marks, pixel intensity along a cross section of a cell was determined using the open source ImageJ distribution package, FIJI [73]. Intensity profiles were smoothed using a moving average with a window size of 5 observations and the findpeaks function from the pracma package (https://CRAN.R-project.org/package=pracma) used to call peaks that increased for at least two successive observations, then decreased for the next 2 observations and exceeded the channel-specific average intensity calculated per cell. Overlapping peaks were determined using the countOverlaps function from the IRanges package [71].

### *Xist* RNA FISH

*Xist* RNA FISH was performed on ES cells, at day 5 of differentiation in our differentiation system, as previously described [6], with modifications. *Xist* RNA was detected with the 15 kb cDNA, pCMV-Xist-PA, as described [72]. The *Xist* probe was labelled with SpectrumGold dUTP (Vysis) by nick translation (Vysis). The cells were mounted in Vectashield HardSet mounting medium with DAPI (Vector Laboratories). Cells were visualised on the LSM 780 fitted with GaAsP detectors (Zeiss) using an α-Plan Apochromat 100X/1.46 oil objective (Zeiss). Images were analysed using the open source ImageJ distribution package, FIJI [73]. For each time point, 100–250 nuclei were analysed.

## Availability of supporting data

All NGS data are deposited in GEO, accession number GSE66526.

## Additional files

The following additional data files are available with the online version of the paper.

10.1186/s13072-016-0064-6 Location of histone ChIPseq peaks in female, male and Setdb1-depleted female MEFs. The genomic location of H3K9me2, H3K9me3, H3K27me3 ChIP-seq peaks (**a-b**) from *Setdb1* ^+/+^ *Xist* ^∆A/+^ 129/CAST MEFs transduced with Nons (Female) (n = 3). Location of genes (purple) and ChIP-seq tracks for each histone mark based on tracks from Seqmonk (www.bioinformatics.babraham.ac.uk/projects/seqmonk/). Peaks called using Seqmonk in-built MACS peak caller shown as grey bars beneath the tracks for one region of the X chromosome (**a**) and the olfactory receptors on chromosome 2 (**b**). The height of the track indicates the read number related to H3, where reads above the axis shows enrichment and below is depletion. Significance of enrichment or depletion is given by the strength of colour – red is most enriched, blue is most depleted. (**c**) Enrichment analysis, showing percentage coverage of genes (black), pseudogenes (grey) and H3K9me2 (green), H3K9me3 (blue) and H3K27me3 (red) ChIP-seq peaks at 50 kb bins along the Female X chromosome. The Pearson correlation coefficient (r) is indicated. (**d**) SNPs between 129 and CAST genomes under peaks of each histone mark on the X chromosome (at left). At right, the average number of H3 reads per peak, for each histone mark, shown as a box-plot.
10.1186/s13072-016-0064-6 Immunofluorescence (IF) detects H3K9 methylation on the inactive X chromosome. (**a**) Representative images showing IF staining for DAPI (grey), H3K9me2 (blue) and as a marker of the Xi Smchd1 (green), in MEFs. A representative histogram showing pixel intensity along a cross-section of the displayed cell is also shown. (**b**) As for **a**, but showing H3K9me3 (pink). The percentage of cells showing overlap in the pixel traces between histone marks and Smchd1 is also shown, as well as p-values determined by Chi^2^ test.
10.1186/s13072-016-0064-6 Schematic of the two variant assays for the reactivation of the X-linked GFP transgene in X_i_^GFP^X_a_ MEFs. MEFs were transduced with shRNA-containing retroviruses and selected with puromycin. Cells were treated with either four doses (**a**) or one dose (**b**) of 10 μM 5-azacytidine. The proportion of GFP positive cells was subsequently determined by flow cytometry and normalised to the non-silencing negative control. n = 4 (**a**) or n = 3 (**b**), mean + s.e.m.
10.1186/s13072-016-0064-6 A retroviral shRNA library used to screen for genes involved in the maintenance of X inactivation. The genes targeted and the numbers of hairpins against each are shown. Dnmt1 [66,67] and Smchd1 [68] hairpins have been previously published.
10.1186/s13072-016-0064-6 H3K9 methyltransferase knockdown validation. qRT-PCR of H3K9 methyltransferase expression levels in cells transduced with H3K9 methyltransferase shRNAs, 3 days after transduction. Results are relative to the average of housekeeping genes *Hmbs* and *Hprt*, then normalised to Nons. n = 3, mean ± s.e.m. One way ANOVA with Dunnett correction for multiple testing. * p < 0.05, ** p < 0.01, *** p < 0.005, **** p < 0.001.
10.1186/s13072-016-0064-6 Failure of in vivo XCI in Setdb1^gt/gt^ female embryos. a. Genomic location (mm10) of the integration site of the gene trap construct. Arrow heads and long arrow indicate the location of oligonucleotides used to identify the integration **b.** Domain structure of wild type Setdb1 protein and the predicted gene trap β-geo fusion protein. **c.** Setdb1 protein expression in wild type and gene trap heterozygote MEFs, as determined by Western blotting (at left), quantified compared to the Actin control (at right). Western blots for H3K9me2, H3K9me3 and H3K27me3 are also shown for the same samples, quantified against tubulin. Bar graphs show quantitation of blots with statistics determined by Student’s one-tailed t-test, *** p < 0.005. (**d**) qRT-PCR of *Setdb1* in primary gene trap heterozygous or wild type MEFs transduced with Nons or *Setdb1* hairpins respectively. Relative to *Hmbs* and normalised to wild type Nons. n = 2, mean ± s.e.m.
10.1186/s13072-016-0064-6 Homozygous lethality of Setdb1^gt/gt^ embryos. Representative bright field images of E7.5 *Setdb1* ^+/+^, *Setdb1* ^gt/+^, and *Setdb1* ^gt/gt^ embryos at 63 × magnification. Embryonic (em), extra-embryonic (ex) and ectoplacental cone (ec) tissues are indicated. Scale bar represents 200 µm.
10.1186/s13072-016-0064-6 Setdb1 peaks genome-wide. The genomic location of Setdb1 ChIP-seq peaks in *Setdb1* ^+/+^ *Xist* ^∆A/+^ 129/CAST MEFs transduced with Nons (Female) (n = 3, **a**) or *Setdb1* ^+/gt^ *Xist* ^∆A/+^ 129/CAST MEFs transduced with shSetdb1.6 (Setdb1 depleted) (n = 2, **b**). (**c**) Example Setdb1 peaks at the Pgk1 and Polrmt genes. (**d**) Metagene analysis of Setdb1 peaks found in Female, Setdb1 depleted or male samples on the X chromosome, relative to WCE control for each genotype.
10.1186/s13072-016-0064-6 Location of histone and Setdb1 ChIP-seq peaks on the Female X chromosome. (**a**) 129/CAST MEFs *Setdb1* ^+/+^ *Xist* ^∆A/+^ transduced with Nons (Female) (n = 3), *Setdb1* ^+/gt^ *Xist* ^∆A/+^ transduced with shSetdb1.6 (Setdb1 depleted) (n = 2) and Setdb1 +/+ XY (Male) (n = 3). Location of genes (purple) and histone peaks as determined by ChIP-seq for H3K27me3 (red), H3K9me2 (green) and H3K9me3 (blue), and Setdb1 (black) on the X in Female, derived from a Seqmonk browser track (www.bioinformatics.babraham.ac.uk/projects/seqmonk/). (**b**) Representative ChIP-seq tracks for H3K9me2, H3K9me3 and H3K27me3.
10.1186/s13072-016-0064-6 DNA methylation is largely unaltered upon Setdb1 depletion outside of X-linked CpG islandseRRBS data from *Setdb1* ^+/+^ *Xist* ^∆A/+^ 129/CAST transduced with Nons (Female) or *Setdb1* ^+/gt^ *Xist* ^∆A/+^ 129/CAST transduced with shSetdb1.6 (Setdb1 depleted) MEFs, showing mC for individual CpGs on the Xa and Xi in Female (n = 3) and Setdb1 depleted female samples (n = 2), for CpGs outside CGIs on the X chromosome (Student’s two-tailed t-test) as log_2_(mC/C) (**a**), within CGIs on autosomes (**b**), outside CGIs on autosomes (**c**) and within CGIs with greater than 20% mC on autosomes (**d**). Note that autosomal data used is not allele specific. Dotted line indicates median for Xi in Female.
10.1186/s13072-016-0064-6 CGIs lose mC upon Setdb1 depletion. Figure shows all the CGIs on the X chromosome that significantly lose mC upon Setdb1 depletion in Female MEFs, when each individual CpG within the CGI is considered. The number of SNPs analysed within each CGI is indicated (n), as is the p-value (p). Mean +s.e.m. Student’s two-tailed t-test. Note that scales are different on all graphs.
10.1186/s13072-016-0064-6 RNA-seq data confirms correct behaviour of the Xist^∆A^ allele. Reads at Xist are shown for the Xi and Xa from both the MEF (**a**) and ES (**b**) cell RNA-seq datasets, showing that the *Xist* ^∆A^ forces non-random XCI in both MEF and ES cells. (c, d) Box plots showing the transcriptional output, expressed as the total average expression from either the Xi or Xa, of cells transduced with shSetdb1.4 (n = 2), shSetdb1.6 (n = 2) and Nons (n = 2), along a time course of differentiation of *Xist* ^∆A/+^ 129/CAST ES cells. Data is shown from replicate 1 (**c**) and 2 (**d**). p-values determined by Student’s two-tailed t-test.
10.1186/s13072-016-0064-6 Reactivation of the inactive PGK X chromosome measured by gene. Aggregated counts for the reference and alternative alleles across all SNPs within a gene. Genes displayed showed p < 0.1 for at least one sample (compared to control, Fisher’s exact test). Significant *p*-values (*p* < 0.05) are in bold.
10.1186/s13072-016-0064-6 Expression of key genes from ES cell RNA-seq. The expression of *Setdb1*, all known H3K9 methyltransferases, representative pluripotency genes (*Nanog*, *Zfp42* and *Tet1*), differentiation gene (*Fgf5*) and *Xist*, expressed as RPKM, for cells transduced with shSetdb1.4 (n = 2), shSetdb1.6 (n = 2) and Nons (n = 2), along a time course of differentiation of *Xist* ^∆A/+^ 129/CAST ES cells. Mean +s.e.m. The number of SNPs analysed within the exons of each gene is indicated (n). Mean +s.e.m. p-values determined by Student’s two-tailed t-test.
10.1186/s13072-016-0064-6 Genes that fail to establish silencing upon Setdb1 depletion. The SNPs that show a failure to establish silencing at day 5 of differentiation in replicates 1 and 2 from RNA-seq in differentiating ES cells are shown. The genes that overlap between replicates 1 and 2 are also shown and the Xi/Xa read count ratio is given.
10.1186/s13072-016-0064-6 LTR repeats are deregulated upon Setdb1 depletion. Analysis of repeat class expression from RNA-seq in differentiating ES cells is shown. Analysis is split into reads mapping either to all chromosomes or specifically to the X chromosome at both days 3 and 5 of differentiation. The average fold-change between Setdb1 knockdown and Nons is indicated for each repeat, as is the average expression for that repeat. Chi squared calculations based on repeats with a log_2_ fold-change greater than 1 are given.
10.1186/s13072-016-0064-6 Primer sequences. List of cloning and qRT-PCR primers used in this study.
10.1186/s13072-016-0064-6 shRNA Sequences. 97-mer shRNA oligonucleotides used to create the retroviral shRNAs for the X inactivation screen and additional hairpins used in this study. The common miR30 5’, loop and 3’ sequences are in black, the unique 21-mer stem sequences are in red and the mismatched bases are in blue.

## Abbreviations

XCI: X chromosome inactivation
Xi: inactive X
Xa: active X
H3K27me3: histone H3 lysine 27 tri-methylation
H3K9me2: histone H3 lysine 9 di-methylation
H3K9me3: histone H3 lysine 9 tri-methylation
Setdb1: set domain bifurcated 1
ES: embryonic stem
PRC2: polycomb repressive complex 2
H2AK119ub1: histone 2A lysine 119 monoubiquitination
MEFs: mouse embryonic fibroblasts
ChIP-seq: chromatin immunoprecipitation followed by sequencing
Dnmt1: DNA methyltransferase 1
Smchd1: structural maintenance of chromosomes hinge domain containing 1
SNPs: single nucleotide polymorphisms
5-aza: 5-azacytidine
Nons: non-silencing
shRNA: short hairpin RNA
CGIs: CpG islands
XIC: X inactivation centre
eRRBS: enhanced reduced representation bisulphite sequencing
NGS: next-generation sequencing
